# Phylogenetic molecular evolution and recombination analysis of complete genome of human parechovirus in Thailand

**DOI:** 10.1038/s41598-021-88124-8

**Published:** 2021-04-21

**Authors:** Thaweesak Chieochansin, Jiratchaya Puenpa, Yong Poovorawan

**Affiliations:** 1 Siriraj Center of Research Excellence for Cancer Immunotherapy, Research Department, Faculty of Medicine Siriraj Hospital, Mahidol University, 2 Prannok Road, Wanglang, Bangkok Noi, Bangkok, 11170 Thailand; 2 Division of Molecular Medicine, Research Department, Faculty of Medicine Siriraj Hospital, Mahidol University, Bangkok, Thailand; 3 Center of Excellence in Clinical Virology, Department of Pediatrics, Faculty of Medicine, Chulalongkorn University, Bangkok, Thailand

**Keywords:** Virology, Molecular evolution, Phylogenetics

## Abstract

Human parechovirus (HPeV), which is a member of the Picornavirus group of viruses, is a pathogen that is reported to be associated with manifestations that include respiratory tract involvement, gastroenteritis, sepsis-like symptom, and central nervous system complication. Until now, nineteen genotypes have been identified. The lack of proofreading property of viral RNA-dependent RNA polymerase (RdRp) together with recombination among the intra- and inter-genotypes of the virus results in high diversity. However, data specific to the molecular evolutionary perspective of the complete genome of HPeV remains limited. This study aimed to analyze the phylogenetic, molecular evolution, and recombination characteristics of the complete genome of HPeV strains isolated in Thailand during 2009–2012. Fifty-eight samples that were previously confirmed to be HPeV positive and then evaluated for genotyping were subjected to complete genome amplification to generate ten overlapping PCR fragments using a set of in-house designed primers. The same position of the viral genome was read in triplicate using direct Sanger sequencing. All samples were classified into the same previously defined genotypes in both whole-genome and VP1 phylogenic tree. However, sample B1091/HPeV14/2011 exhibited discordant grouping between whole-genome and VP1 on the phylogenetic tree. Bootscan analysis revealed that B1091/HPeV14/2011 inherited from two genotypic viruses, including VP1 from HPeV14, and the rest of the genome from HPeV1B. The results of this study provide important insights into the molecular evolution of and recombination in the viral genome of HPeV that will improve and accelerate our ability to develop treatment and prophylactic strategies in the future.

## Introduction

Human parechoviruses (HPeVs) belong to the *Picornaviridea* family, *Parechovirus* genus, *Parechovirus A* species previously described as echoviruses 22 and 23^1–3^. Until now, nineteen genotypes have been classified by the genomic diversity of viral protein (VP) 1, which is assigned as HPeV1-19^1–9^ (http://www.picornastudygroup.com/types/parechovirus/hpev.html). The common manifestation of HPeVs infection is associated with modulated symptoms in the respiratory^10^ and gastrointestinal tract^11,12^, and this virus is mainly found in children^13^. During the past few years, HPeVs have attracted added attention because these viruses were reported to be pathogens that can cause sepsis-like illness and central nervous system (CNS) infection^14^. The epidemiology and clinical manifestations vary according to the genotype of the virus. HPeV1 and HPeV6 were commonly associated with gastroenteritis^15,16^, whereas HPeV3 and HPeV5 were related to more severe manifestations, such as sepsis-like illness^17–20^. Therefore, the molecular epidemiology of these viruses, which leads to genotype identification, is important to investigate.

HPeVs are a nonenveloped virus that exhibit as an icosahedral virion. The virus genome is positive-sense single-stranded RNA (ssRNA), which are approximately 7,300 nucleotides in length. The genome is divided into three parts, including 5′ untranslated region (UTR); polyproteins (P), which consist of P1, P2, and P3; and, 3′UTR. The viral genome translates into a single polyprotein. This translated protein is subsequently cleaved by host protease into three structural proteins, including VP0, VP3, and VP1; and, into seven non-structural proteins, including 2A-C and 3A-D. RNA-dependent RNA polymerase (RdRp), which is encoded from the 3D gene, is responsive for viral genome replication^21,22^. The lack of proofreading property of viral RdRp together with the recombination among intra- and inter-genotypes of the virus leads to high viral genome diversity. The recombination breakpoint hotspots were reported to be around 5′UTR and P1, and P2 and P3 junction^23–27^. However, the molecular evolutionary perspective of the complete genome of HPeV is limited. Therefore, this study aimed to analyze the phylogenetic, molecular evolution, and recombination of the complete genome of HPeVs isolated in Thailand. Together with the reference sequences deposited in the GenBank database, the previously identified viruses from our center during January 2009 to January 2012^28^ were subjected to complete genome amplification to retrieve complete coding sequences and to evaluate their phylogenetic, evolutionary relation recombination event. This study has provided an insightful understanding of molecular evolution and recombination in the viral genome that will pave the way for controlling this virus and for investigating a curative strategy in the future.

## Materials and methods

### Samples and viral genome extraction

All fifty-eight HPeV positive samples were selected during 2009–2012, based on the previous study^28^. Two hundred microliters of 46 fecal samples and 12 nasopharyngeal swab samples were subjected to viral RNA extraction. Extraction was performed using the conventional GTC-phenol–chloroform method^29^. The extracted RNA was finally dissolved in 20 μl of diethylpyrocarbonate (DEPC) water and stored at − 70 °C until further use.

### Complete genome amplification and direct Sanger sequencing

Semi-nested one-step reverse transcription polymerase chain reaction (RT-PCR) was used for amplification. Degenerated primers were designed for ten overlapping PCR fragments based on the GenBank database indicated in Supplementary Table 1. All primers and expected PCR products are shown in Supplementary Table 2. Two microliters of RNA were added to SuperScript™ III One-Step RT-PCR System with Platinum™ Taq DNA Polymerase (Invitrogen Corporation, Carlsbad, CA, USA) for the 1^st^ RT-PCR reaction. Then, two μl of 1^st^ PCR products were used for the second round of semi-nested PCR. PerfectTaq Plus MasterMix (5 PRIME, Darmstadt, Germany) was used as the amplification mixture. The expected PCR products mentioned in Supplementary Table 2 were visualized under ultra-violet light after 2% agarose gel electrophoresis with Tris–Borate-EDTA (TBE) buffer and staining with ethidium bromide. All PCR positive amplicons were purified using Agarose Gelextract Mini Kit (5 PRIME, Darmstadt, Germany). Direct Sanger sequencing was performed by 1^st^ BASE DNA Sequencing Services (1st BASE Laboratories**,** Selangor, Malaysia). Sequencing results were firstly quality check by Chromas Lite (http://www.technelysium.com.au/chromas_lite.html). The contigs with clear chromatogram were then subjected to assembly using SeqMan™ II software (DNASTAR, Madison, WI, USA). Seventy percentage of the sliding window of 100 basepairs were set as the minimal match for assembly parameter. The assembly sequences were further annotated with Basic Local Alignment Search Tool (BLAST) (http://blast.ncbi.nlm.nih.gov/Blast.cgi). The HPeV complete genome's alignment was completed with BioEdit version 7.0.4.1 (Informer Technologies, Inc., Los Angeles, CA, Inc.)^30^.

**Table 1:** The molecular evolution of the HPeV genome.

Genome of HPeV	tMRCA (AD)	95% HPD interval of tMRCA (AD)	Clock rate (substitution/site/year)
Whole genome	1767	1729–1882	1.68 × 10^–3^
VP1	1505	1400–1601	1.56 × 10^–3^
P1	1614	1566–1663	1.86 × 10^–3^
P2	1774	1732–1812	2.21 × 10^–3^
P3	1818	1782–1847	1.96 × 10^–3^

*HPeV* human parechovirus, *tMRCA* the most recent common ancestor, *HPD* highest posterior density, *AD* Anno Domini.

### Molecular evolution analysis

All study sequences were submitted to the GenBank database. The accession numbers included in this study are shown in Supplementary Table 1. Phylogenetic tree, the nucleotide substitution rate, and the most recent common ancestor (tMRCA) were achieved using Bayesian Evolutionary Analysis by Sampling Trees (BEAST) software version 1.10 (University of California, Los Angeles, CA, USA)^31^. The relaxed clock-uncorrelated exponential with 10 million chains was run in the Genetic Testing Registry (GTR) with a gamma distribution substitution model. The data from BEAST were analyzed using the TACER program (http://beast.bio.ed.ac.uk/Tacer). The phylogenetic tree was annotated and analyzed using Figtree version 1.4.4 (http://tree.bio.ed.ac.uk/).

### Recombination analysis

The recombination events were initially analyzed using phylogenetics and genetic distances, after which they were analyzed using the RDP4 software package (http://web.cbio.uct.ac.za/~darren/rdp.html)^32^. The recombination region count matrix, modularity matrix, and recombination breakpoint matrix were generated using RDP4 software in which the default algorithm setting was used. Potential recombination events were also identified by BootScan within RDP4 using a sliding window of 200 nucleotides.

### Ethical consideration

All samples included in this study were sent for routine diagnosis at the Center of Excellence in Clinical Virology of the Faculty of Medicine, Chulalongkorn University, Bangkok, Thailand. Personal data, such as name and hospital number, did not appear in any document relating to this study, including the final manuscript. All samples were taken with permission from the Director of Chulalongkorn King Memorial Hospital, Bangkok, Thailand. Moreover, this study was conducted after receiving approval from the Ethics Committee of the Faculty of Medicine, Chulalongkorn University (IRB approval no.086/53). The Institutional Review Board of the Ethics Committee for Human Research waived the need to obtain written informed consent because all samples were anonymous. All methods were performed in accordance with the relevant guidelines and regulations.

## Results

### Phylogenetic analysis of VP1 and the complete genome of HPeV

**Figure 1:**
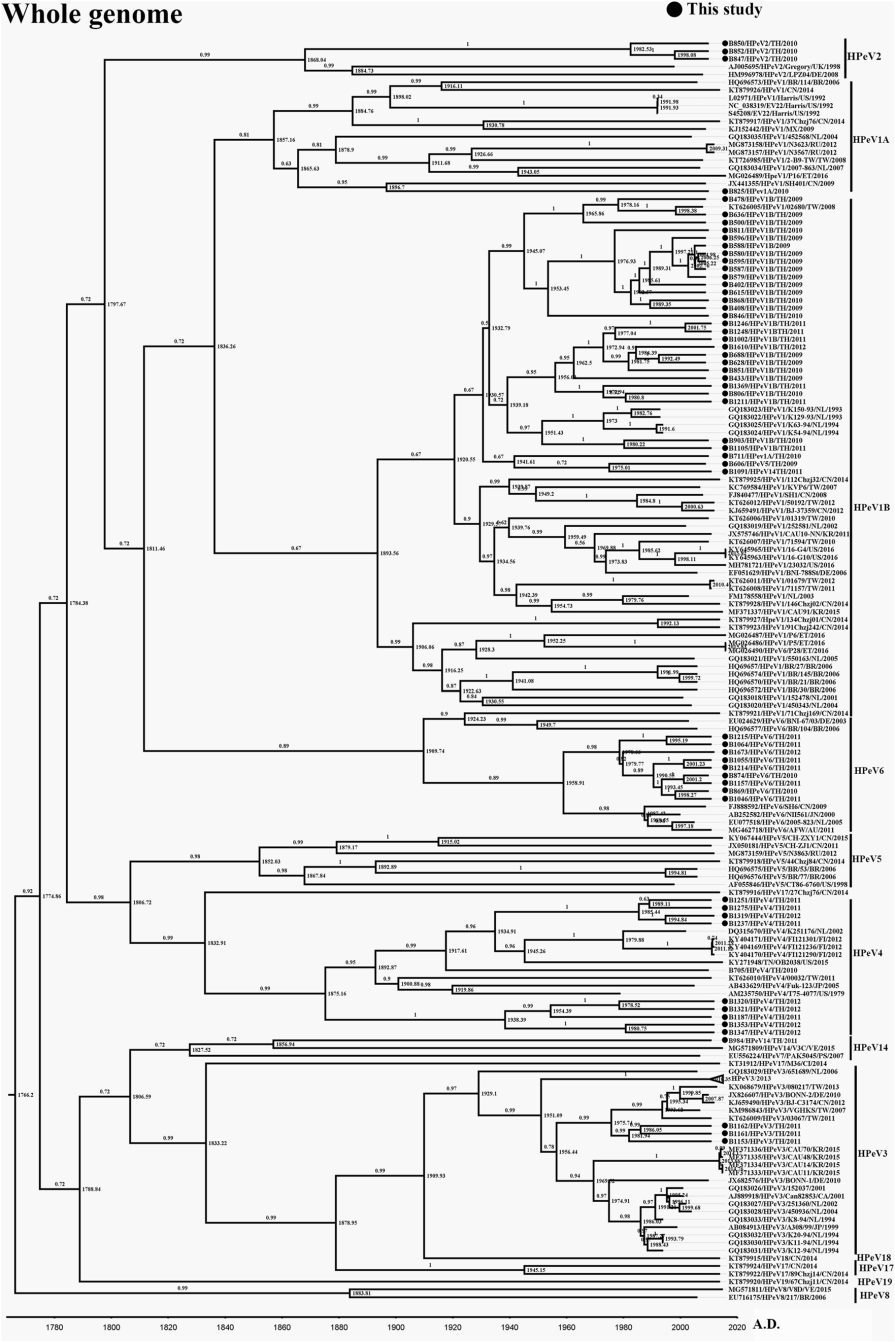
Phylogenetic tree of the complete genome of HPeV. The tree was constructed using the BEAST program under the relaxed clock-uncorrelated exponential with 10 million chains, and was run in the Genetic Testing Registry (GTR) with a gamma distribution substitution model. Each branch is labeled as GenBank accession number/genotype/strain name/origin country/year of collection. The genotype was classified based on the cluster on the VP3/VP1 gene. The samples from this study are indicated with a darkened circle. The most recent common ancestors (tMRCAs) are defined at the tree node, and the highest posterior density (HPD) is indicated at each tree branch.

The viral genome of fifty-eight samples previously identified as HPeV positive^28^ was subjected to complete genome amplification. A set of consensus primers were in-house designed for use in the overlapping semi-nested PCR (Supplementary Table 1). Direct Sanger sequencing revealed the nucleotide sequence of the viral genome after amplification. Each position of a nucleotide was read in at least triplicate with a different direction of sequencing. After assembly and annotation, the complete genomes of HPeVs from this study were evaluated for their genetic correlation with other HPeV strains publicly available in the GenBank database by BEAST program. The sequences from this study were deposited in the GenBank database as accession numbers MW476080 to MW476137. The genotype of viruses classified from this study in the whole-genome phylogenetic tree (Fig. 1) corresponded to the VP1 region (Supplementary Figure S1). However, sample B1091/HPeV14/2011 was classified as genotype 14 from the VP1 sequence (Supplementary Figure S1). In contrast, this sample was grouped with genotype 1B in the whole-genome phylogenetic tree (Fig. 1). This discordant genotype grouping may indicate a recombination event in the virus genome. The year of sample collection was defined as the tip of each taxon. The estimated time to the most recent common ancestor (tMRCA) of the complete genome of the virus indicated as AD 1767 (95% highest posterior density [HPD] interval: 1729–1882) (Table 1). Whereas the tMRCA of VP1 was AD 1505 (95% HPD interval: 1400–1601) (Table 1). The clock rate of VP1 was 1.56 × 10^–3^ substitutions/site/year, whereas the complete genome was 1.68 × 10^–3^ substitution/site/year (Table 1). These clock rates corresponded with the high evolutionary rate in the VP1 region compared with the complete genome.

### Phylogenetic analysis of P1 region of HPeV

**Figure 2:**
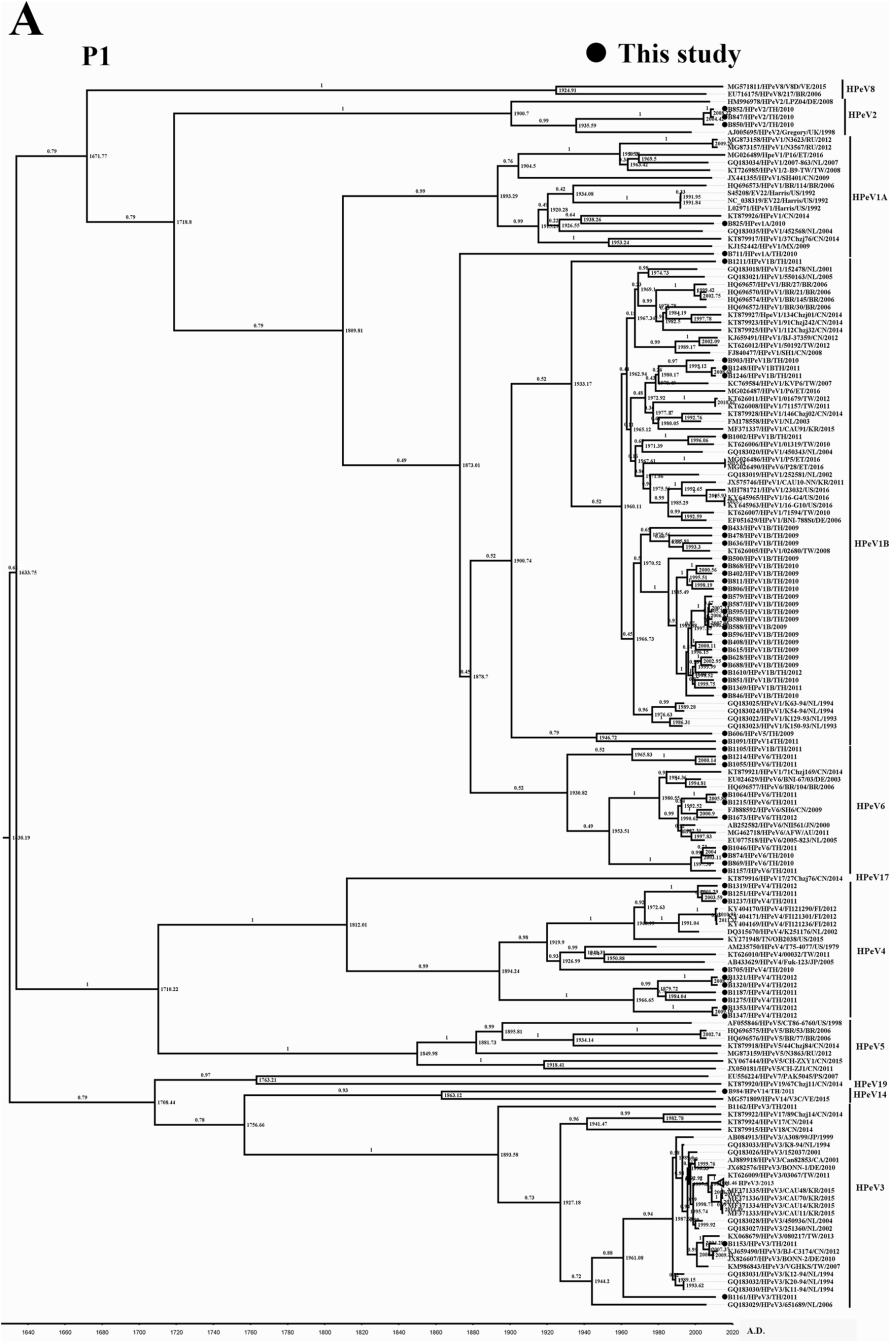

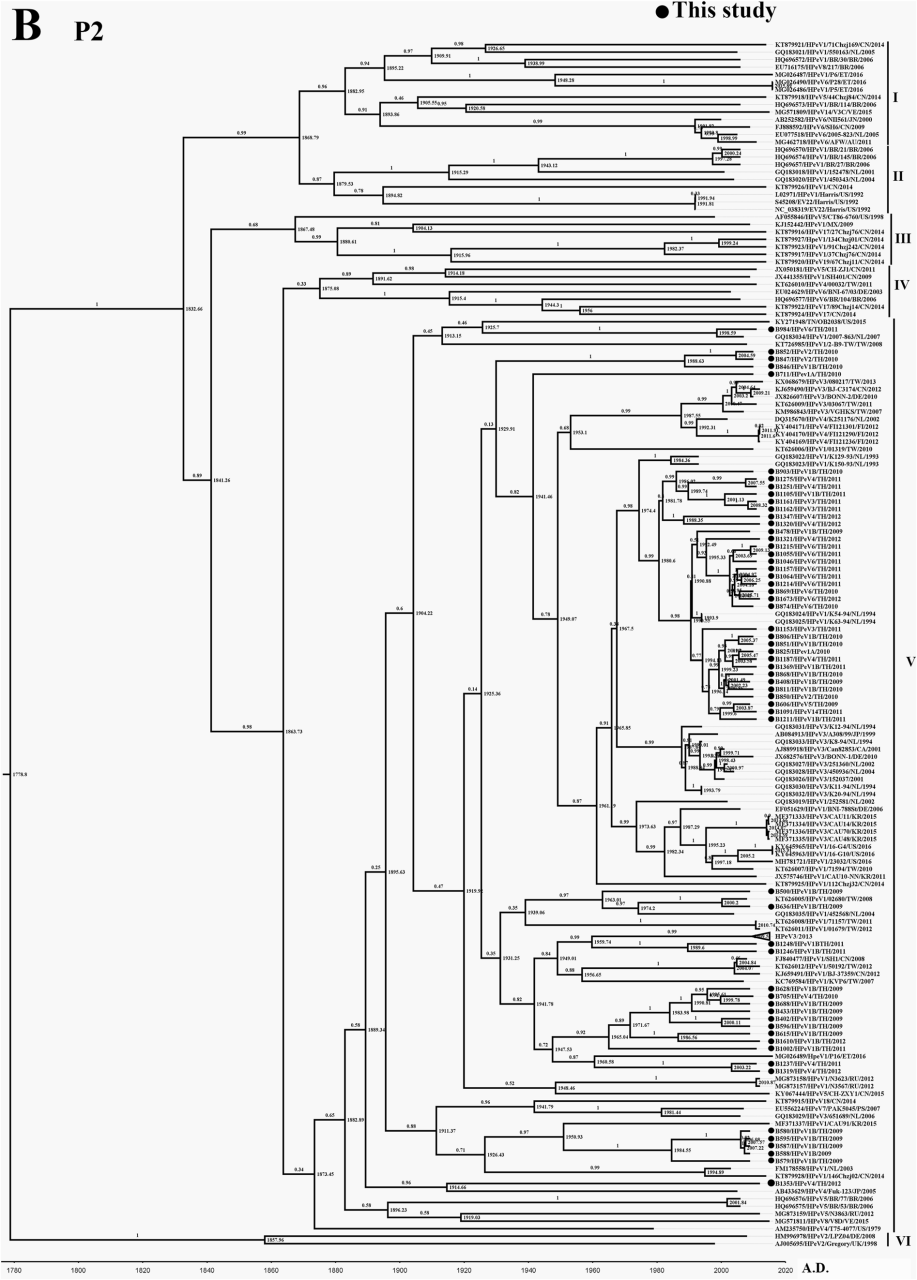

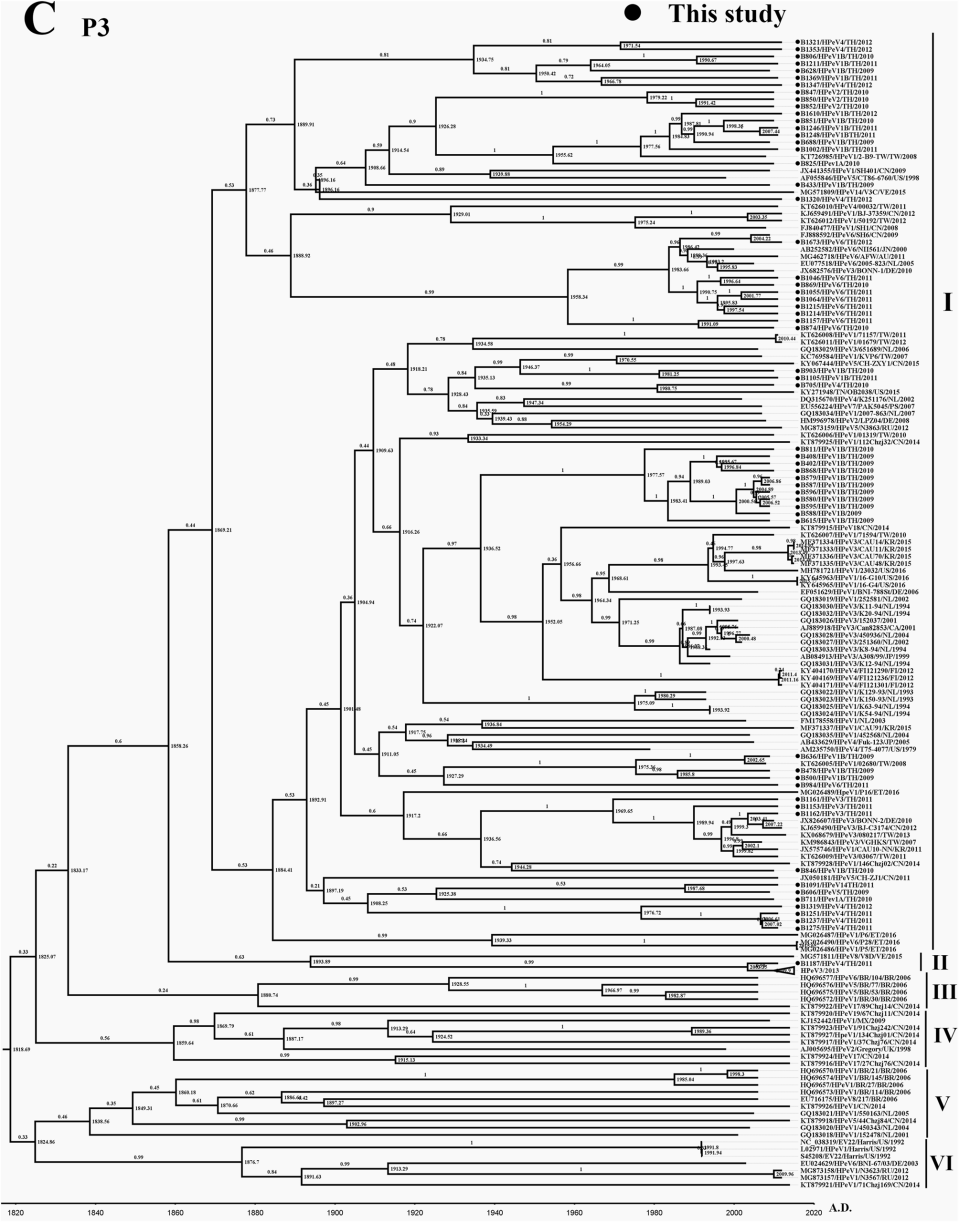
Phylogenetic tree of the P1 (**A**), P2 (**B**), and P3 (**C**) of HPeV. The tree was constructed using the BEAST program under the relaxed clock-uncorrelated exponential with 10 million chains, and was run in the Genetic Testing Registry (GTR) with a gamma distribution substitution model. Each branch is labeled as GenBank accession number/genotype/strain name/origin country/year of collection. The samples from this study are indicated with a darkened circle. The most recent common ancestors (tMRCAs) are defined at the tree node, and the highest posterior density (HPD) is indicated at each tree branch.

**Figure 3:**
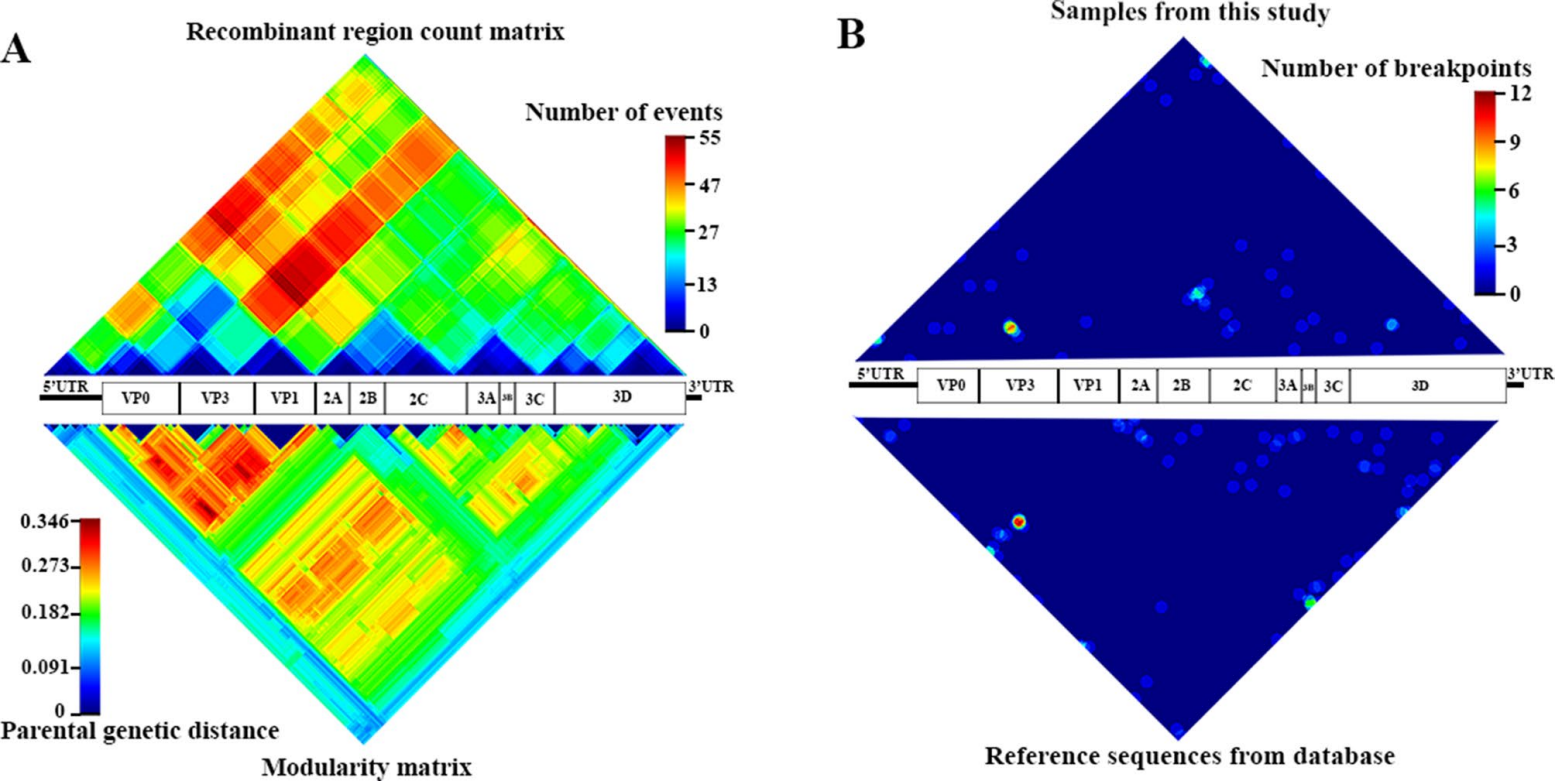
The matrix revealed a recombination event and genetic diversity of the human parechovirus (HPeV) genome. The hot spectrum of recombination region count matrix (upper part of **A**) indicated discordance of inheritance along the entire genome. High genetic diversity has been typically found in the virus's structural protein (hot spectrum of the lower part of **A**). The breakpoint hotspots for recombination in this study appeared in the junctions between VP3 and VP1, and between 2C and 3A (upper matrix of **B**). In comparison, the breakpoint hotspots in the reference sequences that are published in the database are shown between VP1 and 2A (lower matrix of **B**).

The phylogenetic tree of P1 is presented in Fig. 3. Since VP1 is one of the genes incorporated in this region, the genotypes of HPeV defined in P1 were also in concordance with VP1 (Fig. 2A) and the whole-genome phylogenetic tree (Fig. 1). The tMRCA of P1 was AD 1614 (95% HPD interval: 1566–1663), and the clock rate was 1.86 × 10^–3^ substitution/site/year (Table 1). From the phylogenetic tree, three distinct clades were illustrated (Fig. 2A). The first clade comprised five genotypes, including 1A, 1B, 2, 6, and 8. Whereas genotypes 4, 5, and 17 were incorporated into the second clade. Finally, the remaining genotypes (3, 7, 14, and 19) were designated as the third clade (Fig. 2A). tMRCA appraised as AD 1671, 1710, and 1708 in the first, second, and third clade, respectively (Fig. 2A).

### Phylogenetic analysis of P2 and P3 region of HPeV

P2 region of HPeV consists of non-structural proteins 2A, 2B, 2C, and 2D, which are suspected of playing a crucial role in pre-replication and post-translational modification^22^. In comparison, P3 consists of 3A, 3B, 3C, and 3D, which function in the viral replication process^22^. The phylogenetic tree of these two regions of the virus did not group as a genotype as P1 or VP1 region. All genotypes of the virus were unintentionally distributed along the tree of P2 (Fig. 2B) and P3 (Fig. 2C). As previously described by Calvert J., et al. (2010), the minimum of 0.155 nucleotide sequence divergence was the suitable threshold corresponding to a naturally occurring minimum value in the distribution of pairwise distances among 3D sequences^33^. Therefore in this study, the substitution rate over 0.155 was used as the cutoff for classifying the phylogenetic tree of P2 and P3 into different clads. Regarding P2 from this study, six clades of the phylogenetic tree could be defined, including clade I, II, III, IV, V, and VI (Fig. 2B). Interestingly, all samples in this study were members of clade V (the darkened circle in Fig. 2B). tMRCA of P2 was AD 1774 (95% HPD interval: 1732–1812), and the clock rate was 1.56 × 10^–3^ substitutions/site/year (Table 1). In the meantime, the phylogenetic tree of P3 could also be defined into six clades (Fig. 2C). Moreover, all samples from this study were distributed all along with clade I of the tree. It should be noted that only B1187/HPeV4/2011 appeared in clade II (Fig. 2C). tMRCA of P3 was AD 1818 (95% HPD interval: 1782–1847), and the clock rate was 1.96 × 10^–3^ substitution/site/year (Table 1). These results indicate that P2 and P3 occupied less evolutionarily driven rate than the P1 region. All samples isolated from this study were distributed into the same clade, which suggests the regional distribution of P2 and P3 of the virus.

### Recombination investigation in HPeV genome

The recombination analysis of the viral genome was performed using the RDP4 program, after which a consensus event was visualized as a matrix. A recombination event was usually detected in the genome of the viruses included in this study, which indicated in the hot spectrum of recombination region count matrix (upper matrix of Fig. 3A). The result showed that 5′UTR and P1 were inherited from lineage different from that of P2, P3, and 3′UTR. Moreover, the genetic diversity of P1 was also higher than the least of the viral genome, which shows as hot spectrum in the modularity matrix (lower matrix of Fig. 3A). The consensus sequences indicated two hotspots for recombination in the viral genome of our samples. Those breakpoint hotspots appeared in the junction between VP3 and VP1, and between 2C and 3A (upper matrix of Fig. 3B). It may not correspond with the breakpoint in the reference sequences published in the database, which showed only one hotspot between the VP1 and 2A junction (lower matrix of Fig. 3B).

**Figure 4:**
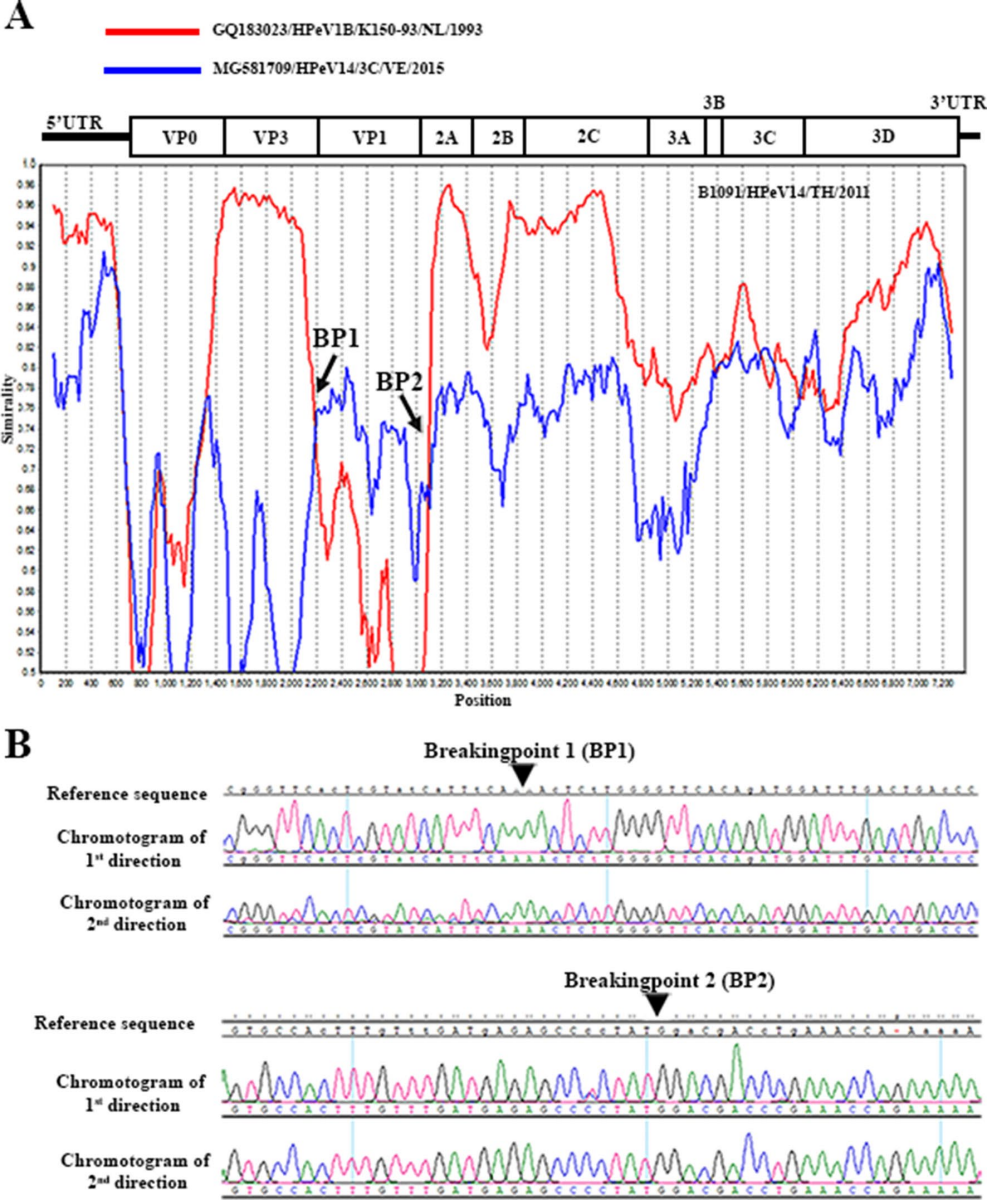
Recombination analysis of B1091/HPeV14/TH/2011. The Bootscan analysis (**A**) was performed using the RDP4 recombination detection program with a sliding window of 200 nucleotides. The result indicated two breakpoints (BPs) between the junctions of VP3 and VP1, and between VP1 and 2A region of the viral genome. These two BPs were confirmed using direct Sanger sequencing (**B**).

In this study, one of the samples (B1091/HPeV14/2011) is suspected of recombination events from the phylogenetic tree analysis (Figs. 2, 3). This genome of the virus seems to be mixed between genotypes 1B and 14. The VP1 region was clustering with genotype 14, whereas the rest of the genome was related to genotype 1B. Therefore, BootScan analysis was applied for suspected recombination examination. The result indicated two breakpoints (BPs) in the genome of B1091/HPeV14/2011 (Fig. 4). BP1 and BP2 were the junctions between VP3 and VP1, and between VP1 and 2A, respectively. The VP1 of the virus was closely related to genotype 14, represented by MG58109/HPeV14/3C/V.E./2015. Meanwhile, the rest of the viral genome was inherited from genotype 1B, represented by GQ183023/HPeV1B/K150-93/NL/1993 (Fig. 4).

## Discussion

HPeV is a member of the *Piconavirudae* family, which was recently reported to be a pathogen related to the respiratory tract, gastrointestinal^11,12^, and central nervous system (CNS) involvement^14^. The lack of proofreading activity of RdRp results in high genetic diversity, which was found in the entire viral genome. Nineteen genotypes have been identified (http://www.picornastudygroup.com/types/parechovirus/hpev.html). The different genotypes of viruses relate to human pathogenesis^22^. By way of example, genotype 3 was closely related to sepsis-like complications^17–20^. Moreover, genetic recombination plays a crucial role in Picornavirus's evolutionary dynamic and diversity. However, this intent analysis in the HPeV genome is very limited. Despite the phylogenetic, molecular evolution, and recombination analysis, it mainly focuses on this study.

Fifty-eight samples previously identified as HPeV positive and that were evaluated for genotyping^28^ were subjected to complete genome amplification. Ten overlapping PCR fragments were performed using a set of in-house designed primers. The exact position of the viral genome was read in triplicate with direct Sanger sequencing. After assembly and annotation, the evolutionary dynamic was revealed with Bayesian's algorithm in BEAST software. Then, the relationship among the different HPeV genotypes from this study was visualized as a phylogenetic tree. All samples were classified into the identical previously defined genotypes in both whole-genome and VP1 phylogenic tree. However, sample B1091/HPeV14/2011 exhibited discordant grouping between whole-genome and VP1 on the phylogenetic tree. Bootscan analysis revealed that B1091/HPeV14/2011 inherited from two genotypic viruses (Fig. 4), including VP1 from HPeV14 (Supplementary Figure S1), and the rest of the genome from HPeV1B (Fig. 1). Inter-genotypic recombination was also reported by Zhao X., et al. (2017), who demonstrated that the virus from fecal isolation (CH-ZXY1) recombined genotypes 1 and 5. Most studies reported that the junction between P1 and P2 was frequency responsiveness for the breakpoint of the HPeV genome^34^ (lower panel of Fig. 3A), and this corresponds with our finding (upper panel of Fig. 3A).

Interestingly, the junction of VP3 and VP1 was another breakpoint indicated in our samples (upper panel of Fig. 3A). Direct Sanger sequencing with triplicate reads was performed to confirm those recombination breakpoints (Fig. 4B). This finding suggested that VP3 and VP1 were other potential points for recombination in the HPeV genome.

HPeV shows dynamic diversity along the genome. High diversity was indicated in the P1 region, which translated into viral structural protein VP1-VP3 (Fig. 2A). In contrast, P2 and P3 of the viral genome exhibited less variation showing in the cold spectrum in Fig. 3. Because the viral structural protein is generally exposed to the environment and is responsive to trigger host immunity, it may be an evolutionarily driven force to increase their variation in the P1 region more than the other viral genes. When we incorporated our samples with the genome in the database, the molecular clock rate of VP1 and P1 was defined as 1.56 × 10^–3^ and 1.68 × 10^–3^ substitutions/site/year, respectively. These rates were slightly lower than those from a previous report by Faria NR., et al. (2009), who reported the substitution rate in VP1 and P1 to be 2.30 × 10^–3^ and 2.03 × 10^–3^ substitutions/site/year, respectively. However, the clock rate of the P3 region, which was 1.96 × 10^–3^ substitutions/site/year (Table 1), was comparable to other reports^35^. Our first report of the evolutionary rate of P2, which was 2.21 × 10^–3^ substitutions/site/year, suggests different evolutionarily driven forces among the HPeV genome.

We also analyzed the complete genome from our isolation with the genome retrieved from the GenBank database, which indicated the root of complete genome sequences as AD 1767 (95% HPD: 1729–1882). This tMRCA was in concordance with the P2 and P3 regions of the virus (Table 1). However, P1 shows as being a bit older than those two regions, indicated as AD 1614 with 95% HPD: 1566–1663, and VP1 was AD 1505 with 95% HPD: 1400–1601. The tMRCAs of P1 and VP1 were comparable to the Faria NR., et al. study, which rooted as AD 1581 with 95% HPD: 1334–1733, and AD 1553 with 95% HPD: 1412–1673, respectively^35^. The overlapping amplification by ten separated PCR to reveal the virus's complete sequence may be the limitation of this study. Only a majority of viral genotypes will be amplified by this method. Next-generation sequencing would be more suitable for complete genome analysis. Unfortunately, in this study, the volume of samples was limited; therefore, we could not subject our samples to the next-generation sequencing process.

## Conclusion

This study successfully retrieved the complete genome of fifty-eight HPeV samples previously isolated from the Thai population^28^. It was clear that one sample (B1091/HPeV14/2011) exhibited a recombination event in which the VP1 gene was inherited from genotype 14, and the rest of the genomes were closely related to HPeV1B. The phylogenetic tree analysis and molecular evolutionary study indicated high diversity at the whole genome level, especially in the P1 region. However, the mechanism of the observed recombination remains unclear and is worthy of further intensive investigation.

## Supplementary Information

Supplementary information.

## References

[1] Wigand R, Sabin AB (1961). Properties of ECHO types 22, 23 and 24 viruses. Arch. Gesamte Virusforsch..

[2] Stanway G (1994). Molecular and biological characteristics of echovirus 22, a representative of a new picornavirus group. J. Virol..

[3] Stanway G, Hyypiä T (1999). Parechoviruses. J. Virol..

[4] Benschop KSM, Williams CH, Wolthers KC, Stanway G, Simmonds P (2008). Widespread recombination within human parechoviruses: analysis of temporal dynamics and constraints. J. Gen. Virol..

[5] Ito M, Yamashita T, Tsuzuki H, Takeda N, Sakae K (2004). Isolation and identification of a novel human parechovirus. J. Gen. Virol..

[6] Oberste MS, Maher K, Pallansch MA (1998). Complete sequence of echovirus 23 and its relationship to echovirus 22 and other human enteroviruses. Virus Res..

[7] Watanabe K, Oie M, Higuchi M, Nishikawa M, Fujii M (2007). Isolation and characterization of novel human parechovirus from clinical samples. Emerg. Infect. Dis..

[8] Li L (2009). Genomic characterization of novel human parechovirus type. Emerg. Infect. Dis..

[9] Drexler JF (2009). Novel human parechovirus from Brazil. Emerg. Infect. Dis..

[10] Harvala H (2008). Epidemiology and clinical associations of human parechovirus respiratory infections. J. Clin. Microbiol..

[11] Zhang DL (2011). Prevalence of human parechovirus in Chinese children hospitalized for acute gastroenteritis. Clin. Microbiol. Infect..

[12] Zhong H (2011). Prevalence and genotypes of human parechovirus in stool samples from hospitalized children in Shanghai, China, 2008 and 2009. J. Med. Virol..

[13] Britton PN, Jones CA, Macartney K, Cheng AC (2018). Parechovirus: an important emerging infection in young infants. Med. J. Aust..

[14] van Hinsbergh TMT, Elbers RG, Hans Ket JCF, van Furth AM, Obihara CC (2020). Neurological and neurodevelopmental outcomes after human parechovirus CNS infection in neonates and young children: a systematic review and meta-analysis. Lancet Child Adolesc. Health.

[15] Malasao R, Khamrin P, Kumthip K, Ushijima H, Maneekarn N (2019). Molecular epidemiology and genetic diversity of human parechoviruses in children hospitalized with acute diarrhea in Thailand during 2011–2016. Arch. Virol..

[16] Pietsch C, Liebert UG (2019). Genetic diversity of human parechoviruses in stool samples, Germany. Infect. Genet. Evol..

[17] Ancora G (2020). Parechovirus infection causing sepsis-like illness in newborns: a NICU approach. New Microbiol..

[18] Boivin G, Abed Y, Boucher FD (2005). Human parechovirus 3 and neonatal infections. Emerg. Infect. Dis..

[19] Harvala H (2009). Specific association of human parechovirus type 3 with sepsis and fever in young infants, as identified by direct typing of cerebrospinal fluid samples. J. Infect. Dis..

[20] Chamings A (2019). An emerging human parechovirus type 5 causing sepsis-like illness in infants in Australia. Viruses.

[21] Harvala H, Wolthers KC, Simmonds P (2010). Parechoviruses in children: understanding a new infection. Curr. Opin. Infect. Dis..

[22] Harvala H, Simmonds P (2009). Human parechoviruses: biology, epidemiology and clinical significance. J. Clin. Virol..

[23] Drexler JF (2011). Full genome sequence analysis of parechoviruses from Brazil reveals geographical patterns in the evolution of non-structural genes and intratypic recombination in the capsid region. J. Gen. Virol..

[24] Benschop K, Thomas X, Serpenti C, Molenkamp R, Wolthers K (2008). High prevalence of human Parechovirus (HPeV) genotypes in the Amsterdam region and identification of specific HPeV variants by direct genotyping of stool samples. J. Clin. Microbiol..

[25] Benschop KS (2010). Comprehensive full-length sequence analyses of human parechoviruses: diversity and recombination. J. Gen. Virol..

[26] Williams CH (2009). Evolution and conservation in human parechovirus genomes. J. Gen. Virol..

[27] Zoll J, Galama JM, van Kuppeveld FJ (2009). Identification of potential recombination breakpoints in human parechoviruses. J. Virol..

[28] Chieochansin T, Vichiwattana P, Korkong S, Theamboonlers A, Poovorawan Y (2011). Molecular epidemiology, genome characterization, and recombination event of human parechovirus. Virology.

[29] Dimke H (2021). Phenol-chloroform-based RNA purification for detection of SARS-CoV-2 by RT-qPCR: comparison with automated systems. PLoS ONE.

[30] Hall TA (1999). BioEdit: A user-friendly biological sequence alignment editor and analysis program for Windows 95/98/NT. Nucl. Acids. Symp. Ser..

[31] Suchard MA (2018). Bayesian phylogenetic and phylodynamic data integration using BEAST 1.10. Virus Evol..

[32] Martin DP, Murrell B, Golden M, Khoosal A, Muhire B (2015). RDP4: Detection and analysis of recombination patterns in virus genomes. Virus Evol..

[33] Calvert J (2010). Recombination dynamics of human parechoviruses: investigation of type-specific differences in frequency and epidemiological correlates. J. Gen. Virol..

[34] Zhao X (2017). The complete genome sequence of a human parechovirus from a child with diarrhea in china revealed intertypic recombination. Genome Announc..

[35] Faria NR, de Vries M, van Hemert FJ, Benschop K, van der Hoek L (2009). Rooting human parechovirus evolution in time. BMC Evol. Biol..

