# Supplementary material for: Phylogenetic molecular evolution and recombination analysis of complete genome of human parechovirus in Thailand
Source: Sci Rep. 2021 Apr 21;11:8572. doi: 10.1038/s41598-021-88124-8 (PMC8060412; doi:10.1038/s41598-021-88124-8)
Supplement: Supplementary file 1 — Supplementary information. [file 41598_2021_88124_MOESM1_ESM.docx]

**Title:** Phylogenetic Molecular Evolution and Recombination Analysis of Complete Genome of Human Parechovirus in Thailand

**Authors:** Thaweesak Chieochansin^1^_’_^2^, Jiratchaya Puenpa^3^, Yong Poovorawan^3^

**Affiliations:**

*^1^Siriraj Center of Research Excellence for Cancer Immunotherapy,* *Research Department, Faculty of Medicine Siriraj Hospital, Mahidol University, Bangkok, Thailand*

*^2^Division of Molecular Medicine, Research Department, Faculty of Medicine Siriraj Hospital, Mahidol University, Bangkok, Thailand*

*^3^Center of Excellence in Clinical Virology, Department of Pediatrics, Faculty of Medicine, Chulalongkorn University, Bangkok, Thailand*

**Corresponding author:**

Thaweesak Chieochansin

Researcher

Siriraj Center of Research Excellence for Cancer Immunotherapy

Research Department

Faculty of Medicine Siriraj Hospital, Mahidol University

2 Prannok Road, Wanglang, Bangkok Noi,

Bangkok 11170, Thailand

Tel: (+66) 2-419-2769

E-mail: [thaweesak.chi@mahidol.ac.th](mailto:thaweesak.chi@mahidol.ac.th).

**Supplementary Table 1.** The list of sequences included in this study

| **GenBank accession number** | **HPeV** | **Remark** |
| --- | --- | --- |
| MW476080 | B711/HPev1A/TH/2010 | From this study |
| MW476080 | B711/HPev1A/TH/2010 | From this study |
| MW476081 | B825/HPev1A/TH/2010 | From this study |
| MW476082 | B402/HPeV1B/TH/2009 | From this study |
| MW476083 | B408/HPeV1B/TH/2009 | From this study |
| MW476084 | B433/HPeV1B/TH/2009 | From this study |
| MW476085 | B478/HPeV1B/TH/2009 | From this study |
| MW476086 | B500/HPeV1B/TH/2009 | From this study |
| MW476087 | B579/HPeV1B/TH/2009 | From this study |
| MW476088 | B580/HPeV1B/TH/2009 | From this study |
| MW476089 | B587/HPeV1B/TH/2009 | From this study |
| MW476090 | B588/HPeV1B/TH/2009 | From this study |
| MW476091 | B595/HPeV1B/TH/2009 | From this study |
| MW476092 | B596/HPeV1B/TH/2009 | From this study |
| MW476093 | B615/HPeV1B/TH/2009 | From this study |
| MW476094 | B628/HPeV1B/TH/2009 | From this study |
| MW476095 | B636/HPeV1B/TH/2009 | From this study |
| MW476096 | B688/HPeV1B/TH/2009 | From this study |
| MW476097 | B806/HPeV1B/TH/2010 | From this study |
| MW476098 | B811/HPeV1B/TH/2010 | From this study |
| MW476099 | B846/HPeV1B/TH/2010 | From this study |
| MW476100 | B851/HPeV1B/TH/2010 | From this study |
| MW476101 | B868/HPeV1B/TH/2010 | From this study |
| MW476102 | B903/HPeV1B/TH/2010 | From this study |
| MW476103 | B1002/HPeV1B/TH/2011 | From this study |
| MW476104 | B1105/HPeV1B/TH/2011 | From this study |
| MW476105 | B1211/HPeV1B/TH/2011 | From this study |
| MW476106 | B1246/HPeV1B/TH/2011 | From this study |
| MW476107 | B1248/HPeV1BTH/2011 | From this study |
| MW476108 | B1369/HPeV1B/TH/2011 | From this study |
| MW476109 | B1610/HPeV1B/TH/2012 | From this study |
| MW476110 | B847/HPeV2/TH/2010 | From this study |
| MW476111 | B850/HPeV2/TH/2010 | From this study |
| MW476112 | B852/HPeV2/TH/2010 | From this study |
| MW476113 | B1153/HPeV3/TH/2011 | From this study |
| MW476114 | B1161/HPeV3/TH/2011 | From this study |
| MW476115 | B1162/HPeV3/TH/2011 | From this study |
| MW476116 | B705/HPeV4/TH/2010 | From this study |
| MW476117 | B1187/HPeV4/TH/2011 | From this study |
| MW476118 | B1237/HPeV4/TH/2011 | From this study |
| MW476119 | B1251/HPeV4/TH/2011 | From this study |
| MW476120 | B1275/HPeV4/TH/2011 | From this study |
| MW476121 | B1319/HPeV4/TH/2012 | From this study |
| MW476122 | B1320/HPeV4/TH/2012 | From this study |
| MW476123 | B1321/HPeV4/TH/2012 | From this study |
| MW476124 | B1353/HPeV4/TH/2012 | From this study |
| MW476125 | B1347/HPeV4/TH/2012 | From this study |
| MW476126 | B606/HPeV5/TH/2009 | From this study |
| MW476127 | B869/HPeV6/TH/2010 | From this study |
| MW476128 | B874/HPeV6/TH/2010 | From this study |
| MW476129 | B984/HPeV6/TH/2011 | From this study |
| MW476130 | B1046/HPeV6/TH/2011 | From this study |
| MW476131 | B1055/HPeV6/TH/2011 | From this study |
| MW476132 | B1064/HPeV6/TH/2011 | From this study |
| MW476133 | B1157/HPeV6/TH/2011 | From this study |
| MW476134 | B1214/HPeV6/TH/2011 | From this study |
| MW476135 | B1215/HPeV6/TH/2011 | From this study |
| MW476136 | B1673/HPeV6/TH/2012 | From this study |
| MW476137 | B1091/HPeV14TH/2011 | From this study |
| NC_038319 | EV22/Harris/US/1992 | For primer designed and phylogenetic analysis |
| S45208 | EV22/Harris/US/1992 | For primer designed and phylogenetic analysis |
| L02971 | HPeV1/Harris/US/1992 | For primer designed and phylogenetic analysis |
| JX575746 | HPeV1/CAU10-NN/KR/2011 | For primer designed and phylogenetic analysis |
| JX441355 | HPeV1/SH401/CN/2009 | For primer designed and phylogenetic analysis |
| JX050181 | HPeV1/BNI-788St/DE/2006 | For primer designed and phylogenetic analysis |
| KY067444 | HPeV5/CH-ZXY1/CN/2015 | For primer designed and phylogenetic analysis |
| EU556224 | HPeV7/PAK5045/PS/2007 | For primer designed and phylogenetic analysis |
| EU716175 | HPeV8/217/BR/2006 | For primer designed and phylogenetic analysis |
| MH781721 | HPeV1/23032/US/2016 | For primer designed and phylogenetic analysis |
| AF055846 | HPeV5/CT86-6760/US/1998 | For primer designed and phylogenetic analysis |
| KY556675 | HPeV3/FEC23/AU/2013 | For primer designed and phylogenetic analysis |
| KY556674 | HPeV3/FEC22/AU/2013 | For primer designed and phylogenetic analysis |
| KY556673 | HPeV3/EC21/AU/2013 | For primer designed and phylogenetic analysis |
| KY556672 | HPeV3/EC20/AU/2013 | For primer designed and phylogenetic analysis |
| KY556671 | HPeV3/CSF19/AU/2013 | For primer designed and phylogenetic analysis |
| KY556670 | HPeV3/AS14/AU/2013 | For primer designed and phylogenetic analysis |
| KY556669 | HPeV3/AS16/AU/2013 | For primer designed and phylogenetic analysis |
| KY556668 | HPeV3/EC17/AU/2013 | For primer designed and phylogenetic analysis |
| KY556667 | HPeV3/EC12/AU/2013 | For primer designed and phylogenetic analysis |
| KY556666 | HPeV3/FEC10/AU/2013 | For primer designed and phylogenetic analysis |
| KY556665 | HPeV3/CSF08/AU/2013 | For primer designed and phylogenetic analysis |
| KY556664 | HPeV3/CSF07/AU/2013 | For primer designed and phylogenetic analysis |
| KY556663 | HPeV3/CSF05/AU/2013 | For primer designed and phylogenetic analysis |
| KY556662 | HPeV3/CSF04/AU/2013 | For primer designed and phylogenetic analysis |
| KY556661 | HPeV3/CSF06/AU/2013 | For primer designed and phylogenetic analysis |
| KY556660 | HPeV3/CSF03/AU/2013 | For primer designed and phylogenetic analysis |
| KY556659 | HPeV3/CSF01/AU/2013 | For primer designed and phylogenetic analysis |
| KY645965 | HPeV1/16-G4/US/2016 | For primer designed and phylogenetic analysis |
| KY645963 | HPeV1/16-G10/US/2016 | For primer designed and phylogenetic analysis |
| KY020128 | HPeV3/SA1/AU/2015 | For primer designed and phylogenetic analysis |
| JX826607 | HPeV3/BONN-2/DE/2010 | For primer designed and phylogenetic analysis |
| JX682576 | HPeV3/BONN-1/DE/2010 | For primer designed and phylogenetic analysis |
| FJ840477 | HPeV1/SH1/CN/2008 | For primer designed and phylogenetic analysis |
| AJ889918 | HPeV3/Can82853/CA/2001 | For primer designed and phylogenetic analysis |
| AM235750 | HPeV4/T75-4077/US/1979 | For primer designed and phylogenetic analysis |
| DQ315670 | HPeV4/K251176/NL/2002 | For primer designed and phylogenetic analysis |
| AB252582 | HPeV6/NII561/JN/2000 | For primer designed and phylogenetic analysis |
| KT726985 | HPeV1/2-B9-TW/TW/2008 | For primer designed and phylogenetic analysis |
| KM986843 | HPeV3/VGHKS/TW/2007 | For primer designed and phylogenetic analysis |
| KJ659491 | HPeV1/BJ-37359/CN/2012 | For primer designed and phylogenetic analysis |
| KJ659490 | HPeV3/BJ-C3174/CN/2012 | For primer designed and phylogenetic analysis |
| FJ888592 | HPeV6/SH6/CN/2009 | For primer designed and phylogenetic analysis |
| GQ183035 | HPeV1/452568/NL/2004 | For primer designed and phylogenetic analysis |
| GQ183034 | HPeV1/2007-863/NL/2007 | For primer designed and phylogenetic analysis |
| GQ183033 | HPeV3/K8-94/NL/1994 | For primer designed and phylogenetic analysis |
| GQ183032 | HPeV3/K20-94/NL/1994 | For primer designed and phylogenetic analysis |
| GQ183031 | HPeV3/K12-94/NL/1994 | For primer designed and phylogenetic analysis |
| GQ183030 | HPeV3/K11-94/NL/1994 | For primer designed and phylogenetic analysis |
| GQ183029 | HPeV3/651689/NL/2006 | For primer designed and phylogenetic analysis |
| GQ183028 | HPeV3/450936/NL/2004 | For primer designed and phylogenetic analysis |
| GQ183027 | HPeV3/251360/NL/2002 | For primer designed and phylogenetic analysis |
| GQ183026 | HPeV3/152037/2001 | For primer designed and phylogenetic analysis |
| GQ183025 | HPeV1/K63-94/NL/1994 | For primer designed and phylogenetic analysis |
| GQ183024 | HPeV1/K54-94/NL/1994 | For primer designed and phylogenetic analysis |
| GQ183023 | HPeV1/K150-93/NL/1993 | For primer designed and phylogenetic analysis |
| GQ183022 | HPeV1/K129-93/NL/1993 | For primer designed and phylogenetic analysis |
| GQ183021 | HPeV1/550163/NL/2005 | For primer designed and phylogenetic analysis |
| GQ183020 | HPeV1/450343/NL/2004 | For primer designed and phylogenetic analysis |
| GQ183019 | HPeV1/252581/NL/2002 | For primer designed and phylogenetic analysis |
| GQ183018 | HPeV1/152478/NL/2001 | For primer designed and phylogenetic analysis |
| AB433629 | HPeV4/Fuk-123/JP/2005 | For primer designed and phylogenetic analysis |
| AB084913 | HPeV3/A308/99/JP/1999 | For primer designed and phylogenetic analysis |
| AJ005695 | HPeV2/Gregory/UK/1998 | For primer designed and phylogenetic analysis |
| KY271948 | TN/OB2038/US/2015 | For primer designed and phylogenetic analysis |
| KT879928 | HPeV1/146Chzj02/CN/2014 | For primer designed and phylogenetic analysis |
| KT879927 | HPeV1/134Chzj01/CN/2014 | For primer designed and phylogenetic analysis |
| KT879926 | HPeV1/CN/2014 | For primer designed and phylogenetic analysis |
| KT879925 | HPeV1/112Chzj32/CN/2014 | For primer designed and phylogenetic analysis |
| KT879924 | HPeV17/CN/2014 | For primer designed and phylogenetic analysis |
| KT879923 | HPeV1/91Chzj242/CN/2014 | For primer designed and phylogenetic analysis |
| KT879922 | HPeV17/89Chzj14/CN/2014 | For primer designed and phylogenetic analysis |
| KT879921 | HPeV1/71Chzj169/CN/2014 | For primer designed and phylogenetic analysis |
| KT879920 | HPeV19/67Chzj11/CN/2014 | For primer designed and phylogenetic analysis |
| KT879918 | HPeV5/44Chzj84/CN/2014 | For primer designed and phylogenetic analysis |
| KT879917 | HPeV1/37Chzj76/CN/2014 | For primer designed and phylogenetic analysis |
| KT879916 | HPeV17/27Chzj76/CN/2014 | For primer designed and phylogenetic analysis |
| KT879915 | HPeV18/CN/2014 | For primer designed and phylogenetic analysis |
| KX068679 | HPeV3/080217/TW/2013 | For primer designed and phylogenetic analysis |
| FM178558 | HPeV1/NL/2003 | For primer designed and phylogenetic analysis |
| KC769584 | HPeV1/KVP6/TW/2007 | For primer designed and phylogenetic analysis |
| EU024629 | HPeV6/BNI-67/03/DE/2003 | For primer designed and phylogenetic analysis |
| MG873159 | HPeV5/N3863/RU/2012 | For primer designed and phylogenetic analysis |
| MG873158 | HPeV1/N3623/RU/2012 | For primer designed and phylogenetic analysis |
| MG873157 | HPeV1/N3567/RU/2012 | For primer designed and phylogenetic analysis |
| MG462718 | HPeV6/AFW/AU/2011 | For primer designed and phylogenetic analysis |
| KY404171 | HPeV4/FI121301/FI/2012 | For primer designed and phylogenetic analysis |
| KY404170 | HPeV4/FI121290/FI/2012 | For primer designed and phylogenetic analysis |
| KY404169 | HPeV4/FI121236/FI/2012 | For primer designed and phylogenetic analysis |
| HM996978 | HPeV2/LPZ04/DE/2008 | For primer designed and phylogenetic analysis |
| HQ696577 | HPeV6/BR/104/BR/2006 | For primer designed and phylogenetic analysis |
| HQ696576 | HPeV5/BR/77/BR/2006 | For primer designed and phylogenetic analysis |
| HQ696575 | HPeV5/BR/53/BR/2006 | For primer designed and phylogenetic analysis |
| HQ696574 | HPeV1/BR/145/BR/2006 | For primer designed and phylogenetic analysis |
| HQ696573 | HPeV1/BR/114/BR/2006 | For primer designed and phylogenetic analysis |
| HQ696572 | HPeV1/BR/30/BR/2006 | For primer designed and phylogenetic analysis |
| HQ69657 | HPeV1/BR/27/BR/2006 | For primer designed and phylogenetic analysis |
| HQ696570 | HPeV1/BR/21/BR/2006 | For primer designed and phylogenetic analysis |
| EU077518 | HPeV6/2005-823/NL/2005 | For primer designed and phylogenetic analysis |
| MG026490 | HPeV6/P28/ET/2016 | For primer designed and phylogenetic analysis |
| MG026489 | HPeV1/P16/ET/2016 | For primer designed and phylogenetic analysis |
| MG026487 | HPeV1/P6/ET/2016 | For primer designed and phylogenetic analysis |
| MG026486 | HPeV1/P5/ET/2016 | For primer designed and phylogenetic analysis |
| MG571811 | HPeV8/V8D/VE/2015 | For primer designed and phylogenetic analysis |
| MG571809 | HPeV14/V3C/VE/2015 | For primer designed and phylogenetic analysis |
| MF371337 | HPeV1/CAU91/KR/2015 | For primer designed and phylogenetic analysis |
| MF371336 | HPeV3/CAU70/KR/2015 | For primer designed and phylogenetic analysis |
| MF371335 | HPeV3/CAU48/KR/2015 | For primer designed and phylogenetic analysis |
| MF371334 | HPeV3/CAU14/KR/2015 | For primer designed and phylogenetic analysis |
| MF371333 | HPeV3/CAU11/KR/2015 | For primer designed and phylogenetic analysis |
| KT626012 | HPeV1/50192/TW/2012 | For primer designed and phylogenetic analysis |
| KT626011 | HPeV1/01679/TW/2012 | For primer designed and phylogenetic analysis |
| KT626010 | HPeV4/00032/TW/2011 | For primer designed and phylogenetic analysis |
| KT626009 | HPeV3/03067/TW/2011 | For primer designed and phylogenetic analysis |
| KT626008 | HPeV1/71157/TW/2011 | For primer designed and phylogenetic analysis |
| KT626007 | HPeV1/71594/TW/2010 | For primer designed and phylogenetic analysis |
| KT626006 | HPeV1/01319/TW/2010 | For primer designed and phylogenetic analysis |
| KT626005 | HPeV1/02680/TW/2008 | For primer designed and phylogenetic analysis |
| KJ152442 | HPeV1/MX/2009 | For primer designed and phylogenetic analysis |

**Supplementary Table 2.** The list of consensus primers used for semi-nested polymerase chain reaction (PCR) to amplify the HPeV genome

| **PCR fragment** | **Primer name** | **Direction** | **Primer sequence**  **(5'-3')** | **Position (5'-3')**  **with L02971** | **Gene** | **~Size in 2^nd^ PCR (bp)** |
| --- | --- | --- | --- | --- | --- | --- |
|  | HPeV-20-OS | Outer Sense | GAR AGC TTG SCC GTM GGG CCT TA | 19-41 | 5**'**UTR | 563 |
| F1 | HPeV-588-IAS | Inner Antisense | CAG ATC CRY AGT GYC DCT TGT TAC C | 606-582 |  |  |
|  | HPeV-660-OAS | Outer Antisense | TCC CCC YYB BRY BTG GCC CRY | 675-655 |  |  |
|  | HPeV- 423-OS | Outer Sense | ACA CGR TGC CYC YGG GGC C | 425-443 | VP0 | 1,068 |
| F2 | HPeV- 447-IS | Inner Sense | GCC RAA AGC CRM GGT TTR RYA RAC C | 440-460 |  |  |
|  | HPeV- 1527-OAS | Outer Antisense | TTY YGR AAR TCH ARY GBA RYA RRC TNC C | 1536-1508 |  |  |
|  | HPeV-1440-OS | Outer Sense | RRA CNG AYT CRT CDG AYY TAG GGC | 1410-1433 | VP3 | 1,048 |
| F3 | HPeV-VP-OAS | Outer Antisense | ACW GTR AAR ATR TCH ACA TSA TDG | 2524-2499 |  |  |
|  | HPeV-VP-IAS | Inner Antisense | DGG YCC ATC ATC YTG WGC TGA | 2458-2438 |  |  |
|  | HPeV-VP-OS | Outer Sense | GAY AAT GCY ATM AYA CWA TYT GTG A | 2090-2115 | VP1 | 961 |
| F4 | HPeV-3231-OAS | Outer Antisense | TCA CAY TCY TCY TCA AYD AYC CAR TCT G | 3231-3204 |  |  |
|  | HPeV-VP-IS | Inner Sense | TTY TCM ACH TGG ATG MGG AAR AC | 2159-2181 |  |  |
|  | HPeV-3117-IAS | Inner Antisense | CCH YCH ACW ATR ATD CCA TAR TGY TT | 3120-3095 |  |  |
|  | HPeV- 2973-OS | Outer Sense | YTN AGR TGY CCH AAY YTN TTY TTY CC | 2936-2961 | 2A-2C | 983 |
| F5 | HPeV- 3945-IAS | Inner Antisense | YTT NAD NAR DGT DTG NAY CCA CCA | 3919-3896 |  |  |
|  | HPeV- 4164-OAS | Outer Antisense | CAR CTR AAR CAN ATD GCC ATD ATD GG | 4140-4115 |  |  |
|  | HPeV- 3792-OS | Outer Sense | GCN GAR RTD GTD GCN GAR TCH ATG TC | 3743-3768 | 2B-3A | 984 |
| F6 | HPeV- 4753-IAS | Inner Antisense | CYC TNC CRT TYT TNG ANA CYT CCC | 4727-4740 |  |  |
|  | HPeV- 4971-OAS | Outer Antisense | YTC CAT YKG YTC RAT NAR NGT NGA CAT | 4948-4922 |  |  |
|  | HPeV- 4679-OS | Outer Sense | TTY CCN TAY ATH ATG CAY ATY MGR GC | 4604-4629 | 3A-3C | 1,115 |
| F7 | HPeV- 5742-IAS | Inner Antisense | RCA CRT NCC TTT RCA DGA YTT NAC NGT | 5719-5693 |  |  |
|  | HPeV- 5817-OAS | Outer Antisense | ATY TCH CCR TTN CCW GCD ATR TGC AT | 5793-5768 |  |  |
|  | HPeV- 5617-OS | Outer Sense | GNW CWG ARA RYA TGY TVA THT GGA TGA C | 5568-5595 | 3C-3D | 972 |
| F8 | HPeV- 6564-IAS | Inner Antisense | TTN ADD GCR YTD ATC ATR AAR TGC | 6540-6517 |  |  |
|  | HPeV- 6600-OAS | Outer Antisense | CCR TCR TAY TGD GAR TAR TCC ATY TCA TA | 6579-6551 |  |  |
|  | HPeV- 3D-OS | Outer Sense | GTN TAY AGR ATG ATH ATG ATG GAR A | 6419-6443 | 3D | 806 |
| F9 | HPeV- 3D-IAS | Inner Antisense | ATN ACM ACW TCA TAA TCA TCC AC | 7221-7199 |  |  |
|  | HPeV- 3D-OAS | Outer Antisense | YTT ART CAA CAC CAT GGG CAY YA | 7253-7230 |  |  |
|  | HPeV- 7053-OS | Outer Sense | TTY CCA GAG TCH ACW TTY ATA GTA GG | 7004-7029 | 3D-3'UTR | 500 |
| F10 | HPeV- 7089-IS | Inner Sense | ACT GAA AAY ATG ATA CAR CAY YTA ATG TGG | 7040-7069 |  |  |
|  | HPeV-3'UTR | Outer Antisense | TTT TTT TTT TTT TTT TTT TTT TTT TTT TTT TTK SBV ADG T | End |  |  |


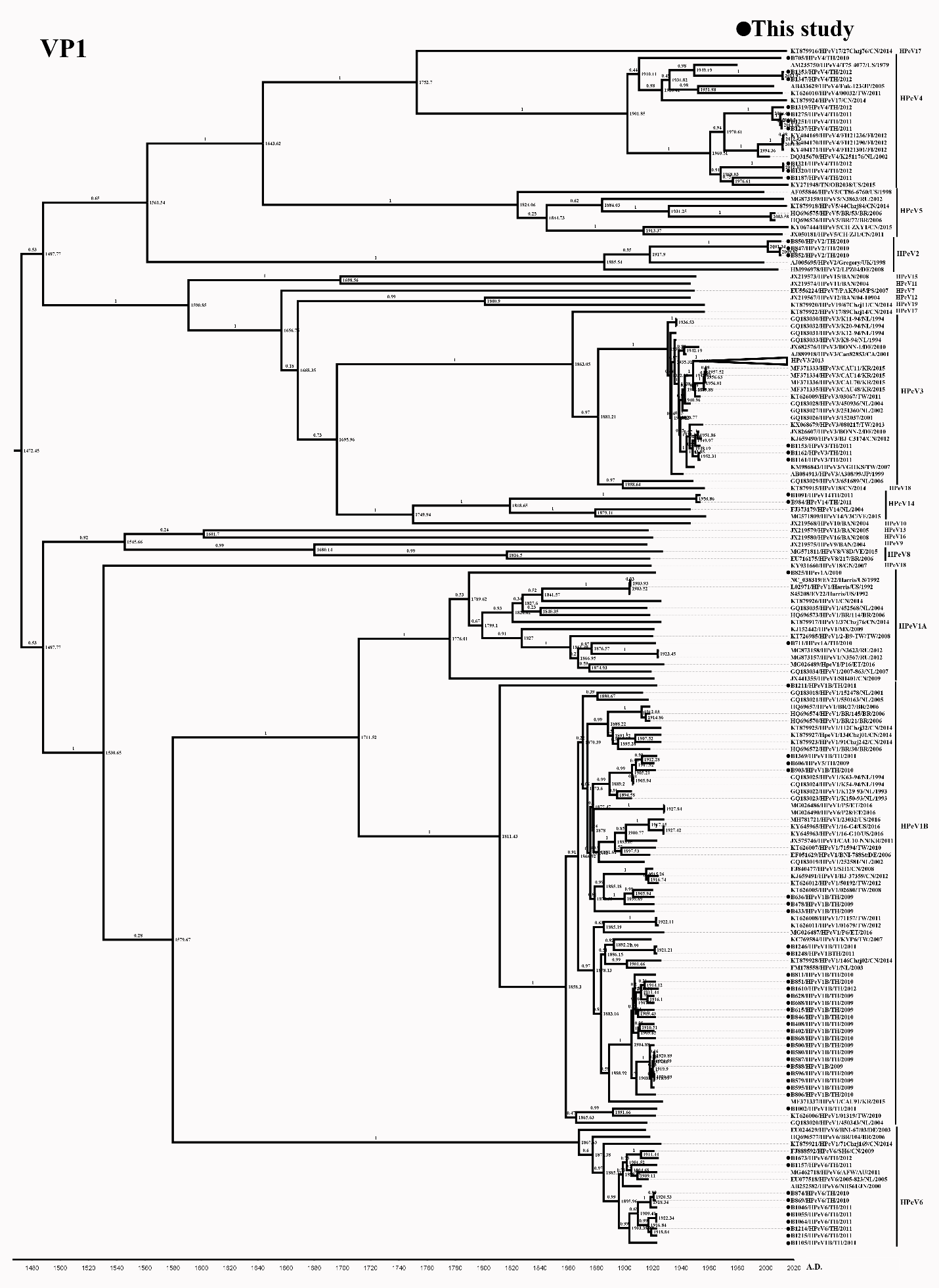


**Supplementary Figure S1.** Phylogenetic tree of the complete VP1 of HPeV. The tree was constructed using the BEAST program under the relaxed clock-uncorrelated exponential with 10 million chains, and was run in the Genetic Testing Registry (GTR) with a gamma distribution substitution model. Each branch is labeled as GenBank accession number / genotype / strain name / origin country / year of collection. The samples from this study are indicated with a darkened circle. The most recent common ancestors (tMRCAs) are defined at the tree node, and the highest posterior density (HPD) is indicated at each tree branch.
